# Autoinducer-2 Plays a Crucial Role in Gut Colonization and Probiotic Functionality of *Bifidobacterium breve* UCC2003

**DOI:** 10.1371/journal.pone.0098111

**Published:** 2014-05-28

**Authors:** Steven E. A. Christiaen, Mary O'Connell Motherway, Francesca Bottacini, Noreen Lanigan, Pat G. Casey, Geert Huys, Hans J. Nelis, Douwe van Sinderen, Tom Coenye

**Affiliations:** 1 Laboratory of Pharmaceutical Microbiology, Ghent University, Ghent, Belgium; 2 Alimentary Pharmabiotic Centre and School of Microbiology, University College Cork, Western Road, Cork, Ireland; 3 Laboratory of Microbiology & BCCM/LMG Bacteria Collection, Ghent University, Ghent, Belgium; University of Ulm, Germany

## Abstract

In the present study we show that *lux*S of *Bifidobacterium breve* UCC2003 is involved in the production of the interspecies signaling molecule autoinducer-2 (AI-2), and that this gene is essential for gastrointestinal colonization of a murine host, while it is also involved in providing protection against *Salmonella* infection in *Caenorhabditis elegans*. We demonstrate that a *B. breve luxS*-insertion mutant is significantly more susceptible to iron chelators than the WT strain and that this sensitivity can be partially reverted in the presence of the AI-2 precursor DPD. Furthermore, we show that several genes of an iron starvation-induced gene cluster, which are downregulated in the *luxS*-insertion mutant and which encodes a presumed iron-uptake system, are transcriptionally upregulated under *in vivo* conditions. Mutation of two genes of this cluster in *B. breve* UCC2003 renders the derived mutant strains sensitive to iron chelators while deficient in their ability to confer gut pathogen protection to *Salmonella*-infected nematodes. Since a functional *luxS* gene is present in all tested members of the genus *Bifidobacterium*, we conclude that bifidobacteria operate a LuxS-mediated system for gut colonization and pathogen protection that is correlated with iron acquisition.

## Introduction

Various beneficial or probiotic effects have been attributed to strains belonging to the genera *Bifidobacterium* and *Lactobacillus*. Probiotic bacteria have been used to treat, among others, antibiotic-associated diarrhea, food allergies, atopic eczema, inflammatory bowel disease and arthritis [1]–[6]. In addition, several studies have inferred a role for probiotic bacteria as antagonists of pathogenic bacteria [7], [8]. Proposed mechanisms of action include competition for the same attachment sites as pathogenic bacteria, competition for nutrients, production of growth-inhibitory compounds and stimulation of the immune system [9]–[12]. Whether probiotics need to adhere to epithelial cells of the human gut in order to exert their beneficial effect is still a matter of debate, but close contact between the two is required at some stage [13]. Bacterial adhesion to the gut epithelium is a complex process in which host, bacterial and environmental factors interact, and it is reasoned that adhesion and associated probiotic activities are regulated by bacterial cell-to-cell communication systems.

Quorum sensing is a cell-to-cell communication system which allows (pathogenic) bacteria to coordinate gene expression and regulate virulence factor production in a cell density-dependent manner [14]–[17]. Many Gram-negative pathogens (e.g. *Pseudomonas aeruginosa*) use N-acylhomoserine lactones as signaling molecules [18]–[21], whereas some Gram-positive bacteria use species-specific oligopeptides [22], [23]. A third cell-to-cell signal molecule is autoinducer-2 (AI-2), produced by a variety of Gram-negative and Gram-positive bacteria. AI-2 is therefore often called an interspecies signaling molecule. A few well-known pathogens, including *Vibrio* spp. and *Salmonella*, use AI-2 as a cue to sense population density [24]–[27]. The key enzyme for AI-2 production is LuxS, which is an essential part of the activated methyl cycle, involved in recycling S-adenosylhomocysteine. More specifically, LuxS catalyzes the cleavage of S-ribosyl-homocysteine to homocysteine and 4,5-dihydroxy-2,3-pentanedione (DPD), which subsequently leads to the production of AI-2 [28]. A wide range of bacterial species produce AI-2, but evidence for the presence of signal reception and signal transduction pathways in organisms besides *Escherichia coli, Vibrio* spp. and *Salmonella* is lacking [29]. While this lack of evidence is sometimes used to question the role of AI-2 in interspecies signaling [30], an alternative explanation is that other types of receptors and signal transduction pathways are yet to be discovered [31].

Although the interspecies signaling molecule AI-2 is commonly linked to virulence and pathogenicity [24]–[27], it has recently been shown that the probiotic strain *Lactobacillus acidophilus* NCFM harbors a functional *luxS* gene and produces AI-2 [32]. Whether this signaling molecule plays a role in eliciting the beneficial traits of probiotic bacteria remains to be determined. Indeed, it was suggested that the ability to produce AI-2 affects attachment of *L. acidophilus* to intestinal epithelial cells, as a mutation in *luxS* was shown to result in decreased adherence to Caco-2 cells [32]. Additionally, *luxS* has been attributed a central metabolic role in *Lactobacillus reuteri* 100–23 and *Lactobacillus rhamnosus* GG, and has been shown to influence adherence, biofilm formation and exopolysaccharide production in the latter [33]–[35]. In a recent study [36] AI-2 production has been demonstrated for three strains of Bifidobacteria and overexpression of *luxS* enhanced biofilm formation by *Bifidobacterium longum* NCC2705.

In the present study we show that a functional *luxS* gene is widespread in the genus *Bifidobacterium* and that this gene in *Bifidobacterium breve* UCC2003 is involved in providing protection of *Caenorhabditis elegans* against *Salmonella* infection, a property which is linked to iron acquisition. Our data furthermore demonstrate that a functional *luxS* gene is required for murine gastrointestinal colonization by *B. breve* UCC2003.

## Methods and Materials

### Bacterial strains, plasmids and culture conditions

The *Bifidobacterium* strains used for the AI-2 biosensor assay are listed in Table 1. These were cultured anaerobically at 37°C in modified Columbia Broth (mCol). Galactose was used to replace glucose as a carbon source since the latter has been reported to possibly interfere with the AI-2 biosensor assay [37], [38]]. All strains were grown until they reached the stationary phase. All other bacterial strains, as well as the plasmids used in this study, are listed in Table 2. *B. breve* UCC2003 and derivative mutant strains were routinely cultured in Reinforced Clostridial Medium (RCM), supplemented with the appropriate antibiotics (10 µg ml^−1^ tetracycline or 3 µg ml^−1^ chloramphenicol). *E. coli* strains were cultured in LB broth at 37°C, while the *V. harveyi* BB170 biosensor strain was grown in Marine Broth at 25°C with agitation [38]. Where appropriate growth media contained tetracycline (Tet; 15 µg ml^−1^), chloramphenicol (Cm; 10 µg ml^−1^ for *E. coli or* 3 µg ml^−1^ for bifidobacteria), erythromycin (Em; 100 µg ml^−1^ for *E. coli*) or kanamycin (Km; 50 µg ml^−1^ for *E. coli*). Recombinant *E. coli* cells containing pORI19 were selected on LB agar containing Em, and supplemented with X-gal (5-bromo-4-chloro-3-indolyl-b-D-galactopyranoside) (40 µg ml^−1^) and 1 mM IPTG (isopropyl-b-D-galactopyranoside).

**Table 1:** List of *Bifidobacterium* strains, with additional information on the source of isolation.

Species	Strain**	Source of isolation	AI-2 production		PCR*
			Mean (%)	SEM (%)	
*B. adolescentis*	LMG 10502^T^	Adult, intestine	226	12	+
	LMG 10733	Adult, intestine	128	5	+
	LMG 10734	Adult, intestine	155	9	+
	LMG 11579	Bovine, rumen	138	7	+
	LMG 18897	Human, faeces	126	5	+
	LMG 18898	Human, faeces	135	16	+
*B. angulatum*	LMG 11039^T^	Human, faeces	129	2	-
	LMG 11568	Sewage	126	12	-
*B. animalis* subsp. *animalis*	LMG 10508^T^	Rat, faeces	237	4	+
	LMG 18900	Rat, faeces	250	11	+
*B. animalis* subsp. *lactis*	LMG 25734	Yoghurt (Yogosan)	242	7	+
	LMG 25755	Yoghurt (Teddi)	207	4	+
	LMG 25756	Food supplement (Hygiaflora)	175	3	+
	LMG 25757	Food supplement (Friendly bifidus)	320	16	+
	LMG 11580	Chicken, faeces	124	6	+
	LMG 18314^T^	Yoghurt	165	3	+
	LMG 18906	Rabbit, faeces	171	5	+
	LMG 23512	Human, faeces	284	10	+
	LMG 24384	Milk	272	23	+
*B. bifidum*	LMG 25758	Pharmaceutical preparation (Infloran Berna)	208	4	+
	LM 381	Food supplement (Friendly bifidus)	447	20	+
	LM 588	Food supplement (Biodophilus)	216	4	+
	LMG 11041^T^	Breast-fed infant, faeces	149	3	+
	LMG 11582	Adult, intestine	152	12	+
	LMG 11583	Adult, intestine	210	7	+
	LMG 13195	Infant, intestine	121	5	+
*B. breve*	LMG 25761	Food supplement (Yakult bifiel)	265	4	+
	UCC2003 (LMG 11040)	Nursing stool	496	41	+
	LMG 11084	Blood	218	4	+
	LMG 11613	Infant, intestine	132	18	+
	LMG 13194	Infant, intestine	447	37	+
	LMG 13208^T^	Infant, intestine	378	14	+
	LMG 23729	Infant, faeces	224	4	+
*B. catenulatum*	LMG 11043^T^	Adult, intestine or faeces	205	4	-
	LMG 18894	Sewage	173	20	-
*B. dentium*	LMG 10507	Human, faeces	126	7	+
	LMG 11045^T^	Dental caries	140	3	+
	LMG 11585	Dental caries	141	11	+
*B. gallicum*	LMG 11596^T^	Adult, intestine	137	3	+
*B. longum* subsp. *infantis*	LMG 25762	Pharmaceutical preparation (Probiotical)	209	4	+
	LMG 8811^T^	Infant, intestine	248	7	+
	LMG 11570	Infant, intestine	152	10	+
	LMG 11588	Infant, faeces	180	5	+
	LMG 13204	Infant, intestine	169	5	+
	LMG 18901	Infant, faeces	119	5	+
	LMG 23728	Infant, faeces	335	13	+
*B. longum* subsp. *longum*	LMG 25765	Yoghurt (Lactoferrin)	260	9	+
	LMG 25766	Food supplement (Lola)	269	9	+
	LMG 11047	Human	121	4	+
	LMG 11589	Calf, faeces	197	5	+
	LMG 13196	Infant, intestine	120	7	+
	LMG 13197^T^	Adult, intestine	207	5	+
	LMG 18899	Adult, faeces	189	19	+
*B. pseudocatenulatum*	LMG 10505^T^	Infant, faeces	189	6	+
	LMG 11593	Sewage	169	17	+
	LMG 18903	Human, faeces	134	4	+
	LMG 18910	Sewage	161	7	+
*B. scardovii*	LMG 21589^T^	50-year-old woman, blood	257	5	+
	LMG 21590	44-year-old woman, hip	260	6	+

For each strain, the relative levels of AI-2 production (means +/- SEM) in the diluted supernatant are given compared to AI-2 levels produced by the biosensor itself ( = 100%). Data presented are mean +/- SEM from triplicate experiments. In addition, the results for the PCR assay with primers directed against *luxS* are shown. (* +, PCR positive result, -, PCR negative result; ** T, type strain of the species).

**Table 2:** Bacterial strains and plasmids used in this study.

Strain or plasmid	Relevant information	Reference or source
Strains		
*V. harveyi* BB170	AI-2 biosensor strain (*luxN*::Tn5)	[38]
* E. coli*		
OP50	*C. elegans* normal food source	Caenorhabditis Genetics Center, University of Minnesota, USA
EC101	Cloning host	[44]
DH5α	AI-2 negative control strain	
* B. breve*		
UCC2003	Wild Type	
UCC2003PK1	UCC2003 harbouring pPKCM	[43]
UCC2003-luxS	Insertion mutant in *lux*S (Bbr_0541)	This study
UCC2003-luxS [pBC1.2luxS]	Complemented strain	This study
UCC2003-bfeU	Insertion mutant in *bfeU* (Bbr_0221)	This study
UCC2003-bfeB	Insertion mutant in *bfeB* (Bbr_0223)	This study
* S. enterica* subsp. *enterica* serovar Typhimurium		
NCTC 13348	Infecting agent	Health Protecting Agency Culture Collections, UK
Plasmids		
pPKCM	pCIBA089-ColE1-Cmr	[43]
pBC1.2	pBC1-pSC101-Cmr	[45]
pORI19	Em^r^, repA^−^, ori^+^, cloning vector	[44]
pAM5	pBC1-puC19-Emr	[45]
pBC1.2luxS	pBC1.2 harboring *luxS* (for complementation)	This study
pORI19-luxS	pOR19 harboring internal fragment of *luxS*	This study
pORI19-luxS-tet	pORI19 harboring internal fragment of *luxS* + Tet^r^	This study
pORI19-bfeU	pOR19 harboring internal fragment of *Bbr_0221*	This study
pORI19-bfeU-tet	pORI19 harboring internal fragment of *Bbr_0221*+ Tet^r^	This study
pORI19-bfeB	pOR19 harboring internal fragment of *Bbr_0223*	This study
pORI19-bfeB-tet	pORI19 harboring internal fragment of *Bbr_0223*+ Tet^r^	This study

### Detection of AI-2 production by Bifidobacteria

A stationary phase culture of a given *Bifidobacterium* strain was centrifuged twice (5,000 g, 5 min, room temperature). Culture supernatant was neutralized (pH 7.0) with 5 M NaOH to exclude any possible pH effects, filter sterilized and subsequently diluted to final concentrations of 20% (v/v) with sterile deionised milliQ water. AI-2 levels were determined in a *V. harveyi* BB170 assay as described previously [38]. Briefly, an overnight culture of the reporter strain was diluted 1∶5000 into fresh sterile, double concentrated MB medium and 100 µl of this cell suspension was added to the wells of a black 96-well microtiter plate (Perkin Elmer). Subsequently, 100 µl of the appropriate sterile supernatant dilution was added to the wells, the microtiter plates were incubated at 30°C and bioluminescence was measured after 5 hours using the EnVision Multilabel Reader (Perkin Elmer). Bioluminescence was expressed as the fraction of bioluminescence measured in the positive control reaction.

### Detection of *luxS* in bifidobacteria

An initial an extensive search of the NCBI Genome Project database (http://www.ncbi.nlm.nih.gov/sites/entrez?db=genome) provided the available sequences of *luxS* homologs in bifidobacteria. Subsequently, a nucleotide BLAST generated a series of additional sequences with high similarity to the sequences found. A set of degenerate primers (5′-CCC GGY TAC ACA TCG ACT GCT C-3′ and 5′-GTG GTC GCG RTA GTT GCC GC-3′) was then designed, using the ClustalX software package as an alignment tool. Extraction of total bifidobacterial bacterial DNA was performed as described previously [39] while PCRs were performed with the following conditions: initial denaturation at 94°C for 3 minutes was followed by 30 cycles of denaturation at 94°C for 30 s, primer annealing at 56°C for 30 s and elongation at 72°C for 30 s. The PCR reactions were terminated with a final elongation of 10 minutes at 72°C. The obtained products were separated by electrophoresis on 1.5% agarose gels and stained with GelRed (Biotium)

### DNA manipulations

The general procedures used for DNA manipulation were essentially those described previously [40]. Restriction enzymes and T4 DNA ligase were obtained from Roche Diagnostics and used according to the manufacturer's instructions. PCRs were performed using Taq PCR master mix (Qiagen GmbH). Synthetic oligonucleotides were synthesized by MWG Biotech AG and are listed in Table S1. PCR products were purified by using a High-Pure PCR product purification kit (Roche). Plasmid DNA was introduced into *E. coli* and *B. breve* by electroporation and large-scale preparation of chromosomal DNA from *Bifidobacterium* spp. was performed as described previously [41]. Plasmid DNA was obtained from *B. breve* and *E. coli* using a QIAprep spin plasmid miniprep kit (Qiagen GmbH). An initial lysis step was performed using 30 mg/ml of lysozyme for 30 min at 37°C as part of the plasmid purification protocol for *B. breve*.

### Construction of *B. breve* UCC2003 insertion mutants and complementation strains

Sequence data were obtained from the Artemis-mediated [42] annotations of the *B. breve* UCC2003 genome [43]. Internal fragments of *luxS* (Bbr_0540, 277-bp), *bfeU* (Bbr_0221, 440 bp) or *bfeB* (Bbr_0223, 457 bp) were amplified by PCR using *B. breve* UCC2003 chromosomal DNA as the template and the oligonucleotide primers luxS-277-f-hindIII and luxS-277-r-xbaI, 221IMhd3 and 221IMxba or 223IMhd3 and 223Imxba, respectively (Table S1). The generated PCR products were cloned into pORI19, an Ori^+^ RepA^−^ integration plasmid [44] using the unique *Hind*III and *Xba*I restriction sites that were incorporated into the forward and reverse primer respectively. Ligations were introduced into *E. coli* EC101 by electroporation. The expected structure of the recombinant plasmids, designated pORI19-luxS, pORI19-bfeU and pORI19-bfeB, was confirmed by restriction mapping and sequencing. The *tet*W gene, amplified by PCR using pAM5 plasmid DNA as the template [45] and primers tetWf and tetWr (Table S1), thereby incorporating flanking *Sal*I sites in the amplicon, was cloned into the *Sal*I-cut pORI19-luxS, pORI19-bfeU and pORI19-bfeB plasmids to generate plasmid pORI19-tet-luxS, pORI19-tet-bfeU and pORI19-tet-bfeB. The latter plasmids were introduced into *E. coli* EC101 harboring pNZ-M.BbrII-M.BbrIII to facilitate methylation [41], and the resulting methylated pORI19-tet-luxS, pORI19-tet-bfeU and pORI19-tet-bfeB were then introduced into *B. breve* UCC2003 by electroporation and subsequent selection on RCA plates supplemented with the tetracycline. Site-specific recombination in potential tet-resistant mutant isolates was confirmed by colony PCR using primer combinations tetWFw and tetWRv to verify tetW gene integration, and primers luxS-Fw, Bbr_0221-Fw and Bbr_0223-Fw (upstream of the *luxS*, *bfeU* and *bfeB* gene fragments selected, respectively), each in combination with pORI19For to confirm integration at the expected chromosomal position. For the construction of the complementation construct pBC1.2luxS, a DNA fragment encompassing *luxS*, including its native promoter region was generated by PCR amplification from chromosomal DNA of *B. breve* UCC2003 using *Pfu* DNA polymerase (Agilent) and primer combination luxS-compl-f-xbaI and luxS-compl-r-xbaI (Table S1). The *luxS*-containing amplicon was digested with *Xba*I, and ligated to similarly digested pBC1.2. The ligation was introduced into *E. coli* EC101 by electroporation. For all cloning experiments, the plasmid content of a number of transformants was screened by restriction analysis and the integrity of positively identified clones was verified by sequencing.

### Transcriptome analysis of *B. breve* UCC2003 and UCC2003-*luxS* during *in vitro* growth

In order to compare global transcription patterns of the *B. breve* UCC2003-luxS insertion mutant with the *B. breve* UCC2003 WT strain, cells grown to early exponential phase were collected and resuspended in DEPC-treated water. Cell disruption, RNA isolation, RNA quality control, cDNA synthesis and indirect labeling were performed as described previously [43]. DNA microarrays containing oligonucleotide primers representing each of the 1,864 annotated genes in the genome of *B. breve* UCC2003 were obtained from Agilent Technologies (Palo Alto). Labeled cDNA was hybridized using the Agilent gene expression hybridization kit (number 5188–5242) as described in the Agilent two-color microarray-based gene expression analysis v4.0 manual (publication number G4140-90050). Following hybridization, microarrays were washed as described in the manual and scanned using Agilent's DNA microarray scanner G2565A. The scans were converted to data files with Agilent's Feature Extraction software (version 9.5). DNA microarray data were processed as previously described [46], [47]. Differential expression tests were performed with the Cyber-T implementation of a variant of the t test [48]. A gene was considered differentially expressed between a mutant and the WT when a transcription ratio of 2 relative to the result for the WT was obtained, with a corresponding p-value equal to or less than 0.01. The transcriptional array data from a dye-swap biological replicate experiment has been deposited in the GEO database under accession number GSE49880.

### Iron chelator assays

The MIC for the iron chelators 2,2-dipyridyl, ciclopirox olamine and phenanthroline was determined according to the European Committee on Antimicrobial Susceptibility Testing (EUCAST) standard broth microdilution protocol, with minor modifications [49]. Instead of Mueller-Hinton broth, RCM was used. A two-fold dilution series of 2,2-dipyridyl, ciclopirox olamine and phenanthroline, ranging from 1000 µM to 2 µM was tested. To assess whether DPD complementation could restore growth of the UCC2003-luxS insertion mutant in the presence of the iron chelators, MICs were also determined in the presence of 50 µM and 100 µM DPD.

### Murine colonization experiments

Experiments with mice were approved by the University College Cork Animal Experimentation Ethics Committee and experimental procedures were conducted under license from the Irish Government (license number B100/3729). Seven-week-old female, BALB/c mice were housed in individually vented cages (Animal Care Systems) under a strict 12 h light cycle. Mice (n = 7 per group) were fed a standard polysaccharide-rich mouse chow diet and water *ad libitum*. Mice were inoculated by oral gavage (10^9^ cfu of *B. breve* UCC2003PK1, *B. breve* UCC 2003-luxS or a mixture of *B. breve* UCC2003PK1 and *B. breve* UCC 2003-luxS in 100 µl of PBS). Fecal pellets were collected at intervals during 18 days to enumerate bacteria. Eighteen days after inoculation, mice were sacrificed and their intestinal tracts quickly dissected. The small intestine, cecum and large intestine were harvested for determination of colony forming units (cfu) (serial dilution plating on RCA agar plates with appropriate antibiotics).

### *C. elegans* colonization experiments

*C. elegans* N2 (*glp-4*; *sek-1*) was propagated under standard conditions, synchronized by hypochlorite bleaching, and cultured on nematode growth medium using *E. coli* OP50 as food source [50], [51].To prepare conditioning plates, WT *B. breve* UCC2003, *B. breve* UCC2003-luxS, *B. breve* UCC2003-luxS [pBC1.2luxS], *B. breve* UCC2003-bfeU or *B. breve* UCC2003-bfeB were grown anaerobically RCM at 37°C until reaching stationary phase. 500 µl of the cell suspension was spread on a nematode growth medium (NGM) plate and dried for 3 h at 37°C [52]. Conditioning plates were used directly after preparation by transferring fresh hypochlorite-treated nematodes to these plates. After conditioning for 72 h, worms were washed three times with M9 buffer supplemented with 1 mM sodium azide to prevent expulsion of the intestinal load and to remove surface-attached bacteria [53]. The number of nematodes was then determined microscopically and nematodes were lysed in phosphate-buffered saline containing 400 mg 1.0 mm silicon carbide beads (BioSpec Products, Inc.) and mechanically disrupted using a pestle. Subsequently, the worm lysates were serially diluted, plated on RCA and incubated anaerobically at 37°C. After 48 h, CFU were determined and the number of bacteria per nematode was calculated [54].

### *In vivo* gene expression analysis by qRT-PCR

A synchronized *C. elegans* nematode population was transferred to conditioning plates (as described above), and after 72 h incubation at 25°C, RNA isolation of *in vivo* grown WT *B. breve* UCC2003 was performed as described previously, with slight modifications [43]. After collection of the nematodes, total RNA was quickly isolated following the protocol of the TRIzol reagent (Invitrogen) and purified using the RNeasy minikit (Qiagen) including an on-column DNase digestion with RNase-free DNase (Qiagen). Next, qScript cDNA Supermix (Quanta Biosciences) was used to obtain cDNA. After development of forward and reverse primers for the reference genes and the genes of interest and after testing their specificity, real-time PCR (CFX96 Real Time 116 6 System, Bio-Rad) was performed using the iQ SYBR Green Supermix (Bio-Rad). The expression levels of the genes of interest were normalized using 5 reference genes, namely, *atp*D, *rpo*B, *ldh*, *pdx*S and *glu*C, by geometric averaging of multiple internal control genes with the GeNorm software package [55]. Primers were designed using Primer-Blast and are listed in Table S1. To ensure the specific amplification of *Bifidobacterium* RNA, primers were also BLASTed to the *C. elegans* and *E. coli* OP50 genome.

### Infection assays in the *C. elegans* model

*C. elegans* survival experiments were performed as described earlier, with slight modifications [56]. Synchronized nematodes (L4 stage) were suspended in a medium containing 95% M9 buffer, 5% brain heart infusion broth, and 10 µg/ml cholesterol (Sigma-Aldrich). Then, 250 µl of this nematode suspension was transferred to the wells of a 24-well microtiter plate. Stationary phase cultures of *E. coli* OP50, *B. breve* UCC2003, *B. breve* UCC2003-luxS, *B. breve* UCC2003-luxS [pBC1.2luxS], *B. breve* UCC2003-bfeU or *B. breve* UCC2003-bfeB were centrifuged, resuspended in the assay medium, and standardized to 10^6^ CFU/ml. Next, 250 µl aliquots of these standardized suspensions were added to each well. Subsequently, 500 µl of the assay medium was added to each well to obtain a final volume of 1 ml per well, and the microtiter plates were incubated at 25°C to allow colonization of the nematode gut. After 24 h incubation, an overnight culture of *S.* Typhimurium NCTC13348 was standardized as described above, and 250 µl of the suspension was added to the wells to establish gastro-intestinal infection. Nematodes not being administered any bifidobacteria were used as a control. Sterile assay medium was added to non-infected nematodes to correct for spontaneous mortality, not caused by *S*. Typhimurium infection. Finally, the plates were incubated at 25°C and the fraction of dead nematodes was determined after 24 h and 48 h by counting the number of dead worms and the total number of worms in each well, using a dissecting microscope.

### Statistical data analysis

Statistical data analysis was carried out using the SPSS Statistics 17.0 software package. To assess if means were statistically significantly different from one another, a non-parametric Mann-Whitney U test was performed (significance level 0.05%).

## Results

### A functional *luxS* gene is widespread amongst bifidobacteria

**Figure 1:**
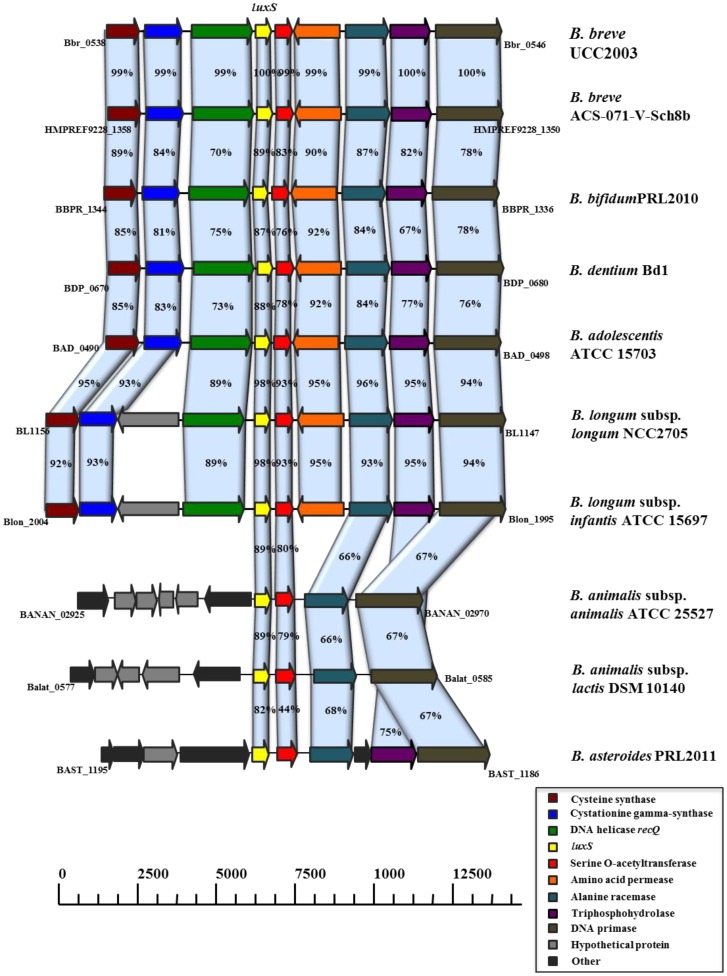
Comparison of the *lux*S genetic loci of *B. breve* UCC2003 with corresponding *lux*S loci from other sequenced bifidobacteria. Each solid arrow indicates an open reading frame. The lengths of the arrows are proportional to the length of the predicted open reading frame. The colour coding which is indicative of putative function, is indicated within the arrow. Orthologs are marked with the same colour while the amino acid identity of each predicted protein is indicated as a percentage relative to its equivalent protein encoded by *B. breve* UCC2003.

The availability of whole genome sequences of various *Bifidobacterium* spp. revealed the presence of the AI-2 synthase-encoding gene *luxS* in these strains. To verify if a functional *luxS* gene is widespread amongst bifidobacteria, phenotypic and genotypic experiments were performed. Using *Vibrio harveyi* BB170 as a biosensor, we demonstrated that all (n = 59) *Bifidobacterium* spp. strains tested produce AI-2 during stationary phase planktonic growth (Table 1). Using primers based on conserved regions of the *luxS* gene we showed that this gene is present in nearly all (n = 55) species investigated, except *Bifidobacterium angulatum* and *Bifidobacterium catenulatum* (Table 1). However, homologs of *luxS* have been found in all currently available genome sequences of *Bifidobacterium* spp. (img.jgi.doe.gov), including those of the two species mentioned above. Inspection of the *lux*S DNA sequence from the currently available genome sequences of *Bifidobacterium angulatum* DSM 20098 (JCM 7096) and *Bifidobacterium catenulatum* LMG 11043 suggests that negative PCR results were due to sequence differences at the PCR primer locations. A map comparing the genomic context of *luxS* in different *Bifidobacterium* genomes is provided in Figure 1 and shows that the organization of *luxS* and its neighboring genes is conserved in the genus *Bifidobacterium*. In particular, the presence and relative genomic position of four genes, encoding a serine O-acetyltransferase, alanine racemase, DNA primase and triphosphohydrolase, are highly conserved (Fig. 1).

**Figure 2:**
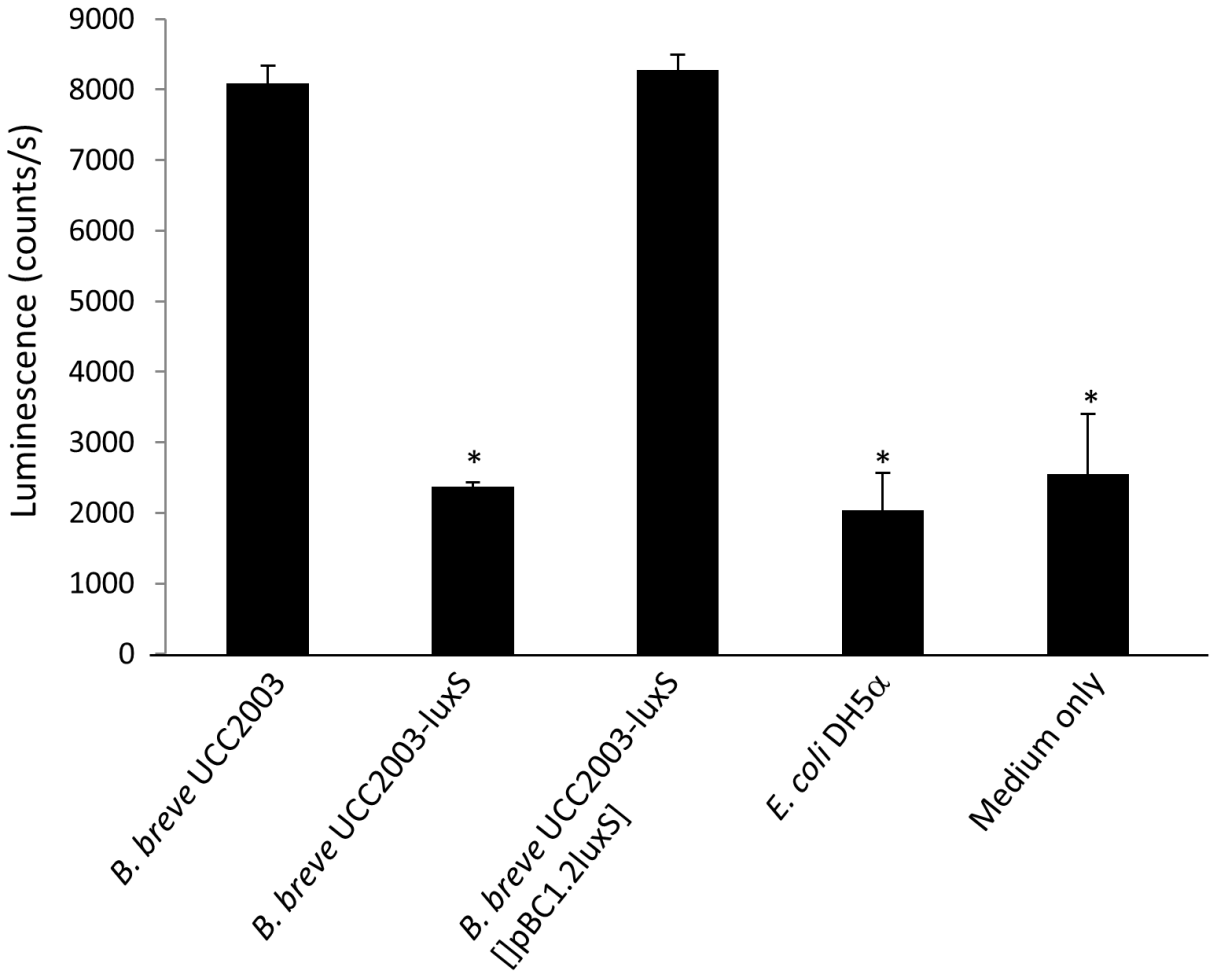
Luminescence signal of the *V. harveyi* BB170 biosensor strain in the presence of sterile and neutralized supernatant of *B. breve* UCC2003, the insertion mutant *B. breve* UCC2003-luxS and the complemented strain *B. breve* UCC2003-luxS [pBC1.2luxS]. Data obtained with *E. coli* DH5α (a strain not producing AI-2) and a medium-only control are included as reference. Data shown are means ± SEM. (*, luminescence is significantly lower than than produced with supernatant of *B. breve* UCC2003, p<0.05, compared to WT; n = 3).

In *B. breve* UCC2003, inactivation of *luxS* by insertional mutagenesis (creating a strain designated as *B. breve* UCC2003-luxS) resulted in a drastic and significant decrease (p≤0.05) in AI-2 production (Fig. 2), thus providing further evidence that *luxS* is crucial for AI-2 production in wild type (WT) UCC2003. This was substantiated by providing a functional *luxS* gene on a plasmid *in trans* in the *luxS*-insertion mutant (strain *B. breve* UCC2003-luxS [pBC1.2luxS]), which restored AI-2 production to WT levels (Fig. 2).

### Impact of *lux*S inactivation on gene expression in *B. breve* UCC2003

To investigate the impact of *luxS* inactivation on gene expression in *B. breve* UCC2003, comparative transcriptome analysis between *in vitro* grown WT *B. breve* UCC2003 and *B. breve* UCC2003-luxS was carried out. The microarray analysis showed that 1.47% of the genes (27/1843) were significantly upregulated in *B. breve* UCC2003-luxS, while 5.70% (105/1843) were significantly downregulated (2-fold cut-off, p≤0.01) compared to the WT. Consistent with previous studies on the role of LuxS in lactobacilli [32]–[35] the microarray analysis suggest that the role of LuxS in *B. breve* is metabolic. Interestingly, the microarray analysis also showed that the transcription of a cluster of six genes, which encode a predicted iron-uptake system (see below) and which had previously been shown to be induced under iron-starvation conditions [57], is downregulated in *B. breve* UCC2003-luxS (Table 3), suggesting that the *luxS* mutation affects the mutant's ability to acquire iron.

**Table 3:** Relative normalized gene expression levels of a cluster of iron regulated genes as expressed *in vitro* in *B. breve* UCC2003-luxS compared to *B. breve* UCC2003.

Locus tag and gene name	Annotation	Fold Downregulation
**Bbr_0221 (bfeU)**	Conserved hypothetical membrane spanning protein with iron permease FTR1 family domain	3.321
**Bbr_0222 (bfeO)**	Conserved hypothetical secreted protein	5.384
**Bbr_0223 (bfeB)**	Conserved hypothetical membrane spanning protein	1.996
**Bbr_0224**	Permease protein of ABC transporter system	5.389
**Bbr_0225**	Permease protein of ABC transporter system	1.730
**Bbr_0226**	ATP-binding protein of ABC transporter system	3.147

### LuxS affects iron metabolism in *B. breve* UCC2003

To explore the possible link between *luxS* and iron metabolism, the tolerance of the *B. breve* strains towards iron chelators that specifically chelate ferrous iron (2,2-dipyridyl), ferric iron (ciclopirox olamine), or both (phenanthroline) was determined. Minimal inhibitory concentration (MIC) values were considerably higher for *B. breve* UCC2003 and *B. breve* UCC2003-luxS [pBC1.2luxS] than that obtained for *B. breve* UCC2003-luxS (Table 4), demonstrating that the insertion mutant is more susceptible to ferrous and ferric ion chelators than WT *B. breve* UCC2003. Addition of the AI-2 precursor DPD to the growth medium partially restored growth of the insertion mutant in the presence of the chelators, thereby supporting the notion that LuxS is directly or indirectly involved in iron acquisition (Table 4). In order to confirm the involvement of the predicted iron-uptake genes in iron acquisition two additional mutant strains, *B. breve* UCC2003-bfeU and *B. breve* UCC2003-bfeB, were constructed that harbor an insertion in the predicted iron-uptake genes, *b*feU (Bbr_0221) and *bfe*B (Bbr_0223), respectively. As expected, both of these mutants were found to be more susceptible to the three iron chelators as compared to the parent strain *B. breve* UCC2003 (Table 4).

**Table 4:** MIC values of 2,2-dipyridyl, ciclopiroxolamine and phenanthroline for *B. breve* UCC2003 WT and various mutants, and for *B. breve* UCC2003-luxS supplemented with DPD (50 µM and 100 µM).

		Component (µM)	
	2,2-dipyridyl	ciclopirox olamine	phenanthroline
***B. breve*** ** UCC2003**	1000	125	250
***B. breve*** ** UCC2003-luxS**	250	31.25	62.5
***B. breve*** ** UCC2003-luxS [pBC1.2luxS]**	1000	125	250
***B. breve*** ** UCC2003-luxS + DPD (50 µM)**	500	62.5	125
***B. breve*** ** UCC2003-luxS + DPD (100 µM)**	500	62.5	125
***B. breve*** ** UCC2003-bfeU**	500	15.63	125
***B. breve*** ** UCC2003-bfeB**	500	31.25	125

### The presence of *luxS* is required for murine gastrointestinal colonization

**Figure 3:**
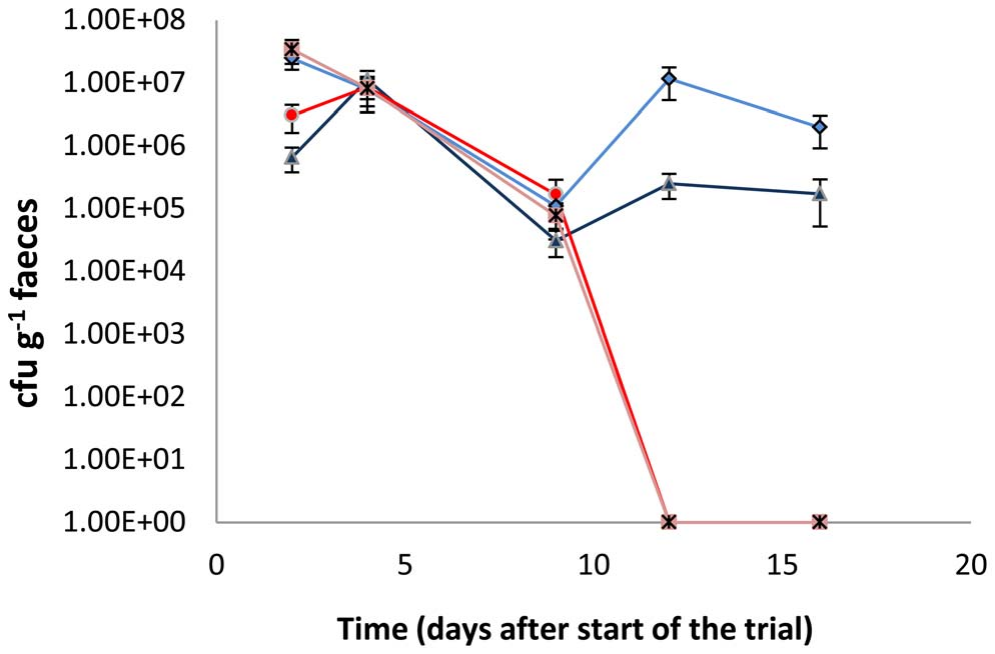
Murine colonization trial. CFU g^−1^ feces of *B. breve* UCC2003 (dark blue) and *B. breve* UCC2003-luxS (red) administered individually, or simultaneously {a mixture of equal numbers of *B. breve* UCC2003 (pale blue) and *B. breve* UCC2003-luxS (pink)}. Administration started at day 0 and was continued for 3 consecutive days. Data shown are mean ± SEM. (n = 7).

To verify whether a functional *luxS* gene is required for gut colonization in a competitive environment, the gut colonization capacity of *B. breve* UCC2003 and *B. breve* UCC2003-luxS was tested in BALB/c mice. In conventional BALB/c mice with a resident microbiota (i.e. in a competitive environment), WT *B. breve* UCC2003 was able to colonize the gastrointestinal tract, as was shown by plating of fecal samples (1×10^5^ CFU/g feces retrieved 15 days after last administration; Fig. 3). Viable count determinations of the contents of the small intestine, large intestine and cecum of individual mice confirmed these findings (Table 5). In contrast, the *luxS* insertion mutant was unable to stably colonize the murine gastrointestinal tract. To examine whether the presence of the WT was able to rescue the *luxS* mutant's impaired ability to colonize, co-administration experiments were carried out. When equal numbers of *B. breve* UCC2003 and *B. breve* UCC2003-luxS were administered simultaneously, only the WT was able to colonize the gastrointestinal tract, once more confirming that a functional *luxS* gene is required for successful colonization of the gastrointestinal tract in a competitive environment (Fig 3).

**Table 5:** Murine colonization experiments.

	LOG CFU retrieved after			
	Single strain administration		Simultaneous administration	
	*B. breve* UCC2003	*B. breve* UCC2003-luxS	*B. breve* UCC2003	*B. breve* UCC2003-luxS
**Small intestine**	6.39±0.09	1.76±1.15 *	5.94±0.43	BDL
**Cecum**	6.87±0.06	2.84±0.67 *	6.96±0.43	BDL
**Large intestine**	6.51±1.34	0.85±1.15 *	6.52±0.43	BDL

LOG CFU of *B. breve* UCC2003 or *B. breve* UCC2003-luxS, retrieved from the murine small intestine, cecum and large intestine (15 days after the last administration). Data are shown for single strain administration as well as for the simultaneous administration of equal numbers of both strains. Numbers shown are means ± SEM. BDL: below detection limit. (*, significantly lower than WT; p<0.05, n = 7).

### *B. breve* UCC2003, *B. breve* UCC2003-luxS or *B. breve* UCC2003-luxS [pBC1.2luxS] colonize the *C. elegans* gut

**Figure 4:**
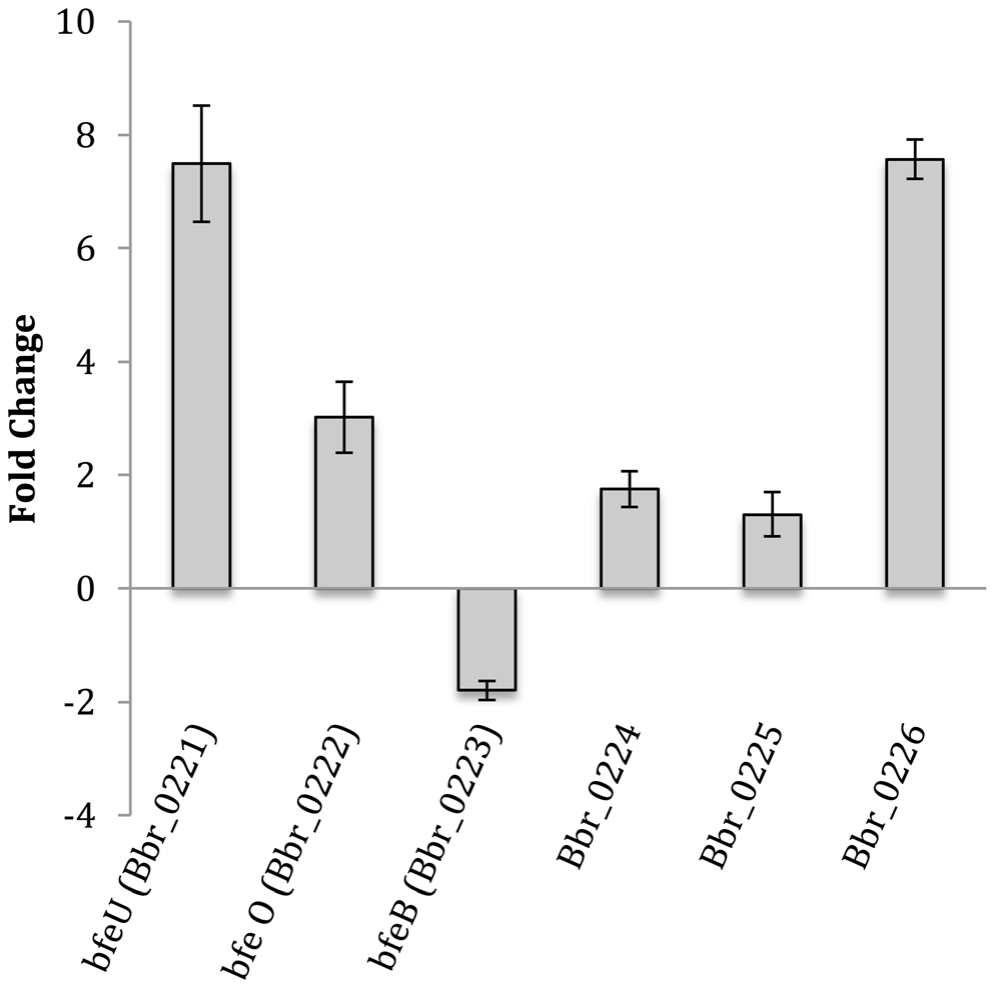
Relative normalized expression levels (obtained with qRT-PCR) of the iron regulated genes in *B. breve* UCC2003 retrieved from *C. elegans* gut, compared to *in vitro* grown *B. breve* UCC2003. Data shown are means ± SEM. (*, p<0.05, compared to *in vitro* expression levels; n = 3).

Having established that UCC2003-luxS could not colonize the murine gastrointestinal tract we sought another model to examine the potential role of *luxS* in providing a host-protecting effect against pathogen infection. We initially determined the colonization capacity of *B. breve* UCC2003, *B. breve* UCC2003-luxS or *B. breve* UCC2003-luxS [pBC1.2luxS] under monoxenic conditions in the gut of the nematode *Caenorhabditis elegans* [58]. When administered separately, the average number of WT *B. breve* UCC2003 able to colonize the *C. elegans* gut was about 1×10^3^ CFU/nematode. The numbers for the insertion mutant *B. breve* UCC2003-luxS (1.20×10^3^ CFU/nematode) and for the complemented strain *B. breve* UCC2003-luxS [pBC1.2luxS] (0.82×10^3^ CFU/nematode) were not significantly different thereby identifying this model as appropriate for infection studies. However, simultaneous administration of a mixture of equal numbers of both WT and *B. breve* UCC2003-luxS revealed that the WT had a competitive advantage, as the average number of CFU recovered were approx. twice as high for the WT (0.81×10^3^ CFU/nematode) as for *B. breve* UCC2003-luxS (0.43×10^3^ CFU/nematode) (p<0.05, n = 3). In order to investigate the importance of the iron-regulated genes for *B. breve* UCC2003 when grown under gastrointestinal conditions in which iron is limited, qRT-PCR experiments were performed whereby the transcription levels of the iron regulated genes was compared between *in vitro* and *in vivo* (i.e. in the *C. elegans* gut) grown *B. breve* UCC2003. These experiments showed that *bfeU* (encoding a high affinity iron permease), *bfeO* (Bbr_0222, encoding a secreted protein with iron-binding domain) and Bbr_0226 (encoding an ABC-type transporter) exhibit a significantly higher level of transcription when *B. breve* UCC2003 was grown under *in vivo* conditions relative to *in vitro* conditions, indicating that in the *C. elegans* gut iron levels are limiting (Fig. 4). These findings are in full agreement with previously obtained *in vivo* transcriptome data (GEO database accession no. GSE27491), showing that six of the seven genes of this iron-regulated cluster also exhibit increased transcription in the murine gut relative to *in vitro* conditions [43].

### *B. breve* confers protection against *Salmonella* infection

**Figure 5:**
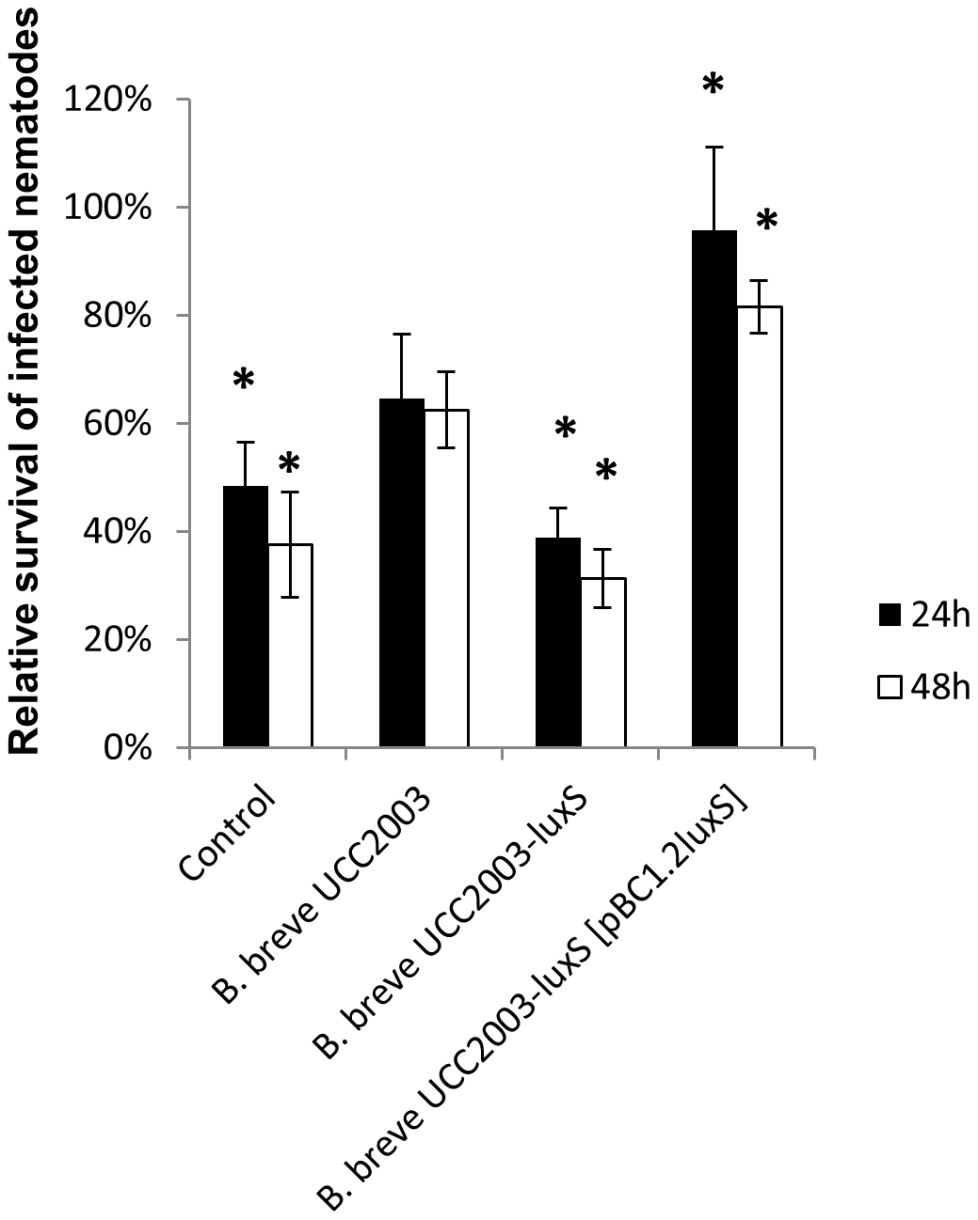
Relative survival of *Salmonella*-infected *C. elegans* nematodes to which *B. breve* UCC2003 WT and various mutants were administered (24 h{black bar} and 48 h{white bars} after *Salmonella* infection). Data shown are means ± SEM. Control: infected nematodes that have not been administered any bifidobacteria. (*  =  significantly increased or decreased survival as compared to *Salmonella*-infected *C. elegans* nematodes to which *B. breve* UCC2003 WT was administered, p<0.05; n = 3).

**Figure 6:**
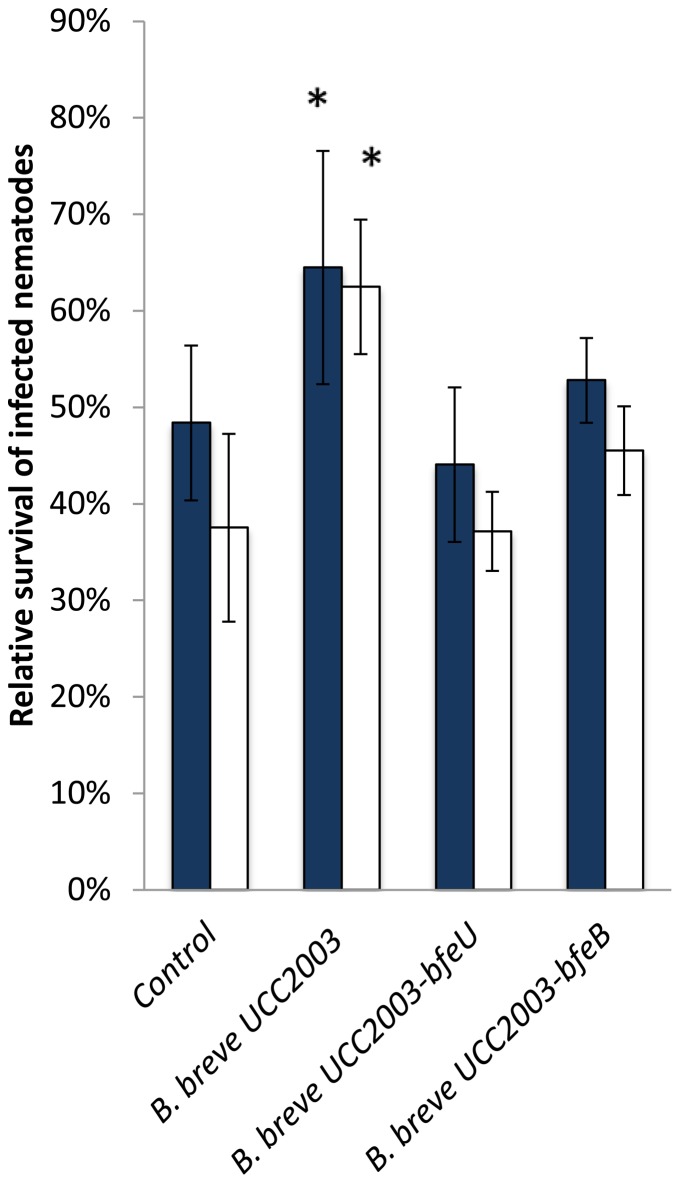
Relative survival of *Salmonella*-infected *C. elegans* nematodes to which *B. breve* UCC2003 WT or mutants UCC2003-bfeU or UCC2003-bfeB were administered (24 h {blue bars} and 48 h {white bars} after *Salmonella* infection). Data shown are means ± SEM. Control: infected nematodes that have not been administered any bifidobacteria. (*  =  significantly different as compared to the control, p<0.05; n = 3).

In order to examine the potential role of *luxS* in providing a host-protecting effect against pathogenic bacteria, an *in vivo Salmonella* infection experiment in the *C. elegans* model was adopted. A positive influence of bifidobacterial administration on the longevity of *Salmonella*-infected nematodes has previously been described [59]. The relative survival of *Salmonella*-infected *C. elegans* worms that were administered *B. breve* UCC2003, *B. breve* UCC2003-luxS or *B. breve* UCC2003-luxS [pBC1.2luxS] is shown in Figure 5. Relative survival of infected nematodes that were fed UCC2003 was significantly higher than survival of the infected nematodes that had not received *B. breve* UCC2003 (p≤0.05). Administration of the *luxS* insertion mutant resulted in a significantly decreased survival of infected nematodes compared to those that had received WT *B. breve* UCC2003, whereas administration of the complemented strain resulted in a significantly increased survival compared to those nematodes that had not received treatment and to those that had received the *luxS* mutant (p≤0.05). Similar to UCC2003-*luxS*, *B. breve* UCC2003-bfeU and *B. breve* UCC2003-bfeB each exhibit a significantly decreased ability to confer protection to *Salmonella*-infected nematodes as compared to the WT *B. breve* UCC2003, thereby confirming the importance of iron acquisition in gut pathogen protection (Fig. 6). Furthermore, as both *B. breve* UCC2003-bfeU and *B. breve* UCC2003-bfeB colonized *C. elegans* to a similar level as the WT, this decreased protective effect is not due to decreased ability to colonize the nematodes. Collectively this data indicates that *luxS* (and its gene product) is a prerequisite for *B. breve* UCC2003 to confer protection against *Salmonella* infection, and that this protection can be correlated with iron acquisition.

## Discussion

Members of the genus *Bifidobacterium* are recognized as being numerically dominant representatives of the microbiota of healthy breast-fed infants [60], [61]. Colonization of the newborn infant gut commences during and after birth by microbes from the mother and the environment. A succession in the gut colonization of healthy vaginally-delivered infants has been reported, whereby initial colonization is by facultative anaerobes that include enterobacteria, staphylococci and streptococci. Once available oxygen in the gut is consumed the newly created anaerobic environment supports establishment of strict anaerobes of the genera *Clostridium, Bacteroides* and *Bifidobacterium* [62]. While this microbiota is recognized as optimal for healthy infants, an in depth understanding of molecular players involved in gut colonization and host protection by such bacteria, in particular *Bifidobacterium* sp., remains to be elucidated. The intricacies of host-microbe interactions in early life cannot be underestimated and the precise mechanisms by which elements of the infant gut microbiota contribute to health maintainence and promotion in early life are only beginning to emerge [63], [64].

The bacterial interspecies signaling molecule AI-2 is now well recognized for its role in the regulation of virulence factor production in pathogenic Gram-negative and Gram-positive bacteria [65]. These virulence-associated features include biofilm formation, toxin production, adherence to epithelial cells, motility as well as the metabolism of heavy metals and carbon [24]–[26]. In addition, the AI-2 synthase LuxS plays an important role in central metabolism, more specifically in the detoxification and recycling of S-adenosylhomocysteine [28]. Interestingly, all *Bifidobacterium* strains sequenced to date harbor a *lux*S gene and our investigations demonstrating that all tested bifidobacterial strains, representing 11 species of this genus, were capable of producing AI-2 is consistent with previous finding of [36] who detected AI-2 production for two Bifidobacterial species. In this respect it seemed intriguing that a gut commensal, dominant in the infant microbiota, would produce a molecule that potentially promotes the production of virulence factors in (opportunistic) gastrointestinal pathogens.

To further investigate this, *B. breve* UCC2003 was chosen as a representative of probiotic bifidobacteria. This strain, in addition to producing high levels of AI-2 in the biosensor assay, is a generally accepted model for the genus *Bifidobacterium* [8], [66]. Transcriptome analysis of *B. breve* UCC2003-luxS versus UCC2003, grown under *in vitro* conditions, revealed that the role of LuxS is primarily metabolic. These findings are supported by the fact that no AI-2 signal transduction pathways have previously been described in bifidobacteria and that a protein with high similarity to the known AI-2 receptor LuxP has not been identified from the genome of *B. breve* UCC2003. UCC2003-luxS was shown to be more sensitive to various iron chelators, and unable to colonize the murine gastrointestinal tract, while this mutant also conferred less protection against *Salmonella* infection in a *C. elegans* nematode model. These data demonstrate that LuxS plays a crucial role for bifidobacteria in their ability to establish themselves as gut commensals, which also includes their beneficial effect pertaining to pathogen protection/exclusion. Furthermore, our results show that LuxS activity is involved in iron acquisition, and we propose that this property gives *B. breve* UCC2003 a competitive advantage in iron-limited environments such as the gastrointestinal tract. The importance of iron acquisition mediated nutritional immunity in gut pathogen protection was further demonstrated by the fact that two additionally constructed mutants harboring insertions in either of two presumed iron-uptake genes proved to have a decreased ability to confer protection against *Salmonella* infection in the *C. elegans* model. In addition and as expected, these mutants were more susceptible to the iron chelators as compared to the parent strain *B. breve* UCC2003. It has previously been shown that LuxS affects genes involved in iron metabolism in *Porphyromonas gingivalis* [67], *Vibrio vulnificus* [68], *Mannheimia haemolytica* [69] and *Actinobacillus pleuropneumoniae* [70], while it was also demonstrated that iron availability increases the pathogenic potential of several gastrointestinal pathogens including *S*. Typhimurium, *Citrobacter freundii*, *E. coli* [71] and *Listeria monocytogenes* [72], [73]. Our results are consistent with the notion that bifidobacteria confer gut pathogen protection by nutritional immunity. This in turn suggests that LuxS/AI-2 can be versatile in various bacterial species and conditions. Since the colonization capacity of a (putative) probiotic bacterium is considered to be a prerequisite to exert its beneficial effects, the observation that AI-2-expressing *B. breve* UCC2003 outcompetes an isogenic derivative lacking this capacity contributes to the elucidation of molecular players and mechanisms of colonization requirements and probiotic effects [74].

Indeed, one application where administration of bifidobacterial strains may positively influence health is in the prevention of necrotizing enterocolitis (NEC) in premature infants. The precise causative agent of NEC is unknown; however, the preterm infant microbiota has been found to be dominated by pathogenic genera with *Proteobacteria* and *Enterobacteriaceae* dominating rather than characteristic species belonging to *Bacteroidetes, Clostridium* and *Bifidobacterium* [75]–[77]. The dominance of potentially pathogenic bacteria may increase the risk of infection in this vulnerable group. Neonatal nurseries in Finland, Italy and Japan have been routinely and successfully using probiotics as prophylaxis against NEC for over a decade, without ever reporting any adverse effects. Despite the safe use of practice and numerous randomized clinical trials that indicate that probiotics can reduce the incidence of NEC by at least 30% [78]–[80], clinical guidelines by the American Society for Parenteral and Enteral Nutrition (A.S.P.E.N) express the view that there is currently insufficient data to recommend the use of probiotics in infants at risk of NEC [81]. Integral to the resistance to adopt probiotics as a prophylaxis against NEC in premature infants is the lack of knowledge on the mechanism of action [82]. While the data presented here is merely one molecular mechanism and one probiotic attribute that is conserved among all bifidobacteria, this research provides a key insight into a mechanism of gut pathogen protection conferred by bifidobacteria that is of clinical relevance.

## Supporting Information

Table S1 **Oligonucleotide primers used in this study**
(DOCX)
