# Supplementary material for: Autoinducer-2 Plays a Crucial Role in Gut Colonization and Probiotic Functionality of Bifidobacterium breve UCC2003
Source: PLoS One. 2014 May 28;9(5):e98111. doi: 10.1371/journal.pone.0098111 (PMC4037206; doi:10.1371/journal.pone.0098111)
Supplement: Table S1 — Oligonucleotide primers used in this study (DOCX) [file pone.0098111.s001.docx]

**Supporting Information Table S1.** Oligonucleotide primers used in this study

| **Purpose** | **Primer** | **Sequence (5'-3')^a^** |
| --- | --- | --- |
|  |  |  |
| Degenerate primers for amplification *luxS* | luxS-fw | Cccggytacacatcgactgctc |
|  | luxS-rev | Gtggtcgcgrtagttgccgc |
|  |  |  |
| Insertional mutation in Bbr_0541 (*luxS*) | luxS-277-f-hindIII | tgcgga**aagctt**gatcacaccaaggtcaag |
|  | luxS-277-r-xbaI | ctatgc**tctaga**gagtgcgtgccccaggtc |
| Cloning of *luxS* in pBC1 | luxS-compl-f-xbaI | ctatgc**tctaga**cattgcctatagcgaatc |
|  | luxS-compl-r-xbaI | ctatgc**tctaga**gcaatcaccagtacaggaac |
| Amplification of TetR gene | tetWf | tcagctgtcgacatgctcatgtacggtaaggaagca |
|  | tetWr | gcgacggtcgaccataacttctgattgttgccg |
| Insertional mutation in Bbr_0221(*bfeU*) | 221IMhd3 | ctgcgc**aagctt**gaatgacgcctactaccagcac |
|  | 221IMxba | ttgacg**tctaga**caccgaacgcgaccatgaag |
| Insertional mutation in Bbr_0223(*bfeB*) | 223IMhd3 | ctgcgc**aagctt**catcgacatcgcgctcaccctgttc |
|  | 223IMxba | ttgacg**tctaga**gaatgccttgtgtttgcgtc |
| Confirmation of site specific  homologous recombination | pORI19R | Attgtgagcggataacaatttcac |
|  | luxS-fw | Tacgtgcgttacattgac |
|  | Bbr_0221 -fw | cgatgcttccgattctg |
|  | Bbr_0223 -fw | cacaaggccatcatgcacatc |
| qRT-PCR primers | Bbr_0997-fw | caagccgttcgctcgcttcc |
|  | Bbr_0997-rev | ggaggcgaagcggtcttggt |
|  | Bbr_1478-fw | tggcatcggagaatgccgagg |
|  | Bbr_1478-rev | acccgcaggtgtcgcctatg |
|  | Bbr_1479-fw | tcggtagcgtcgcccttgtc |
|  | Bbr_1479-rev | tcgagctggccgtttccgag |
|  | Bbr_1377-fw | tcggttccgcactcggctac |
|  | Bbr_1377-rev | cggtctgaatcggctcgcca |
|  | Bbr_1898-fw | catcggcggtgttggtcagc |
|  | Bbr_1898-rev | ggcgacaacccgtacaagcg |
|  | Bbr_1589-fw | aacgccagtgcggcgatgat |
|  | Bbr_1589-rev | gggcgttcatggcaccgttc |
|  | Bbr_1291-fw | aggtggacgtatccgccgtc |
|  | Bbr_1291-rev | ggaaatcgcggacaccgtgg |
|  | Bbr_0563-fw | gcgatgctgcggatagcacc |
|  | Bbr_0563-rev | gcgttcaccggctcgatcac |
|  | Bbr_1042-fw | gcactcagcgccggaatagc |
|  | Bbr_1042-rev | tggcgttcctatggtgccgt |
|  | Bbr_0788-fw | gtgccgggactgaccgactc |
|  | Bbr_0788-rev | gttcgcgagtagcggcgttg |
|  | Bbr_0541-fw | gatcacaccaaggtcaag |
|  | Bbr_0541-rev | gagtgcgtgccccaggtc |
|  | Bbr_1381-fw | ttgcacgaagctgttgacgtattcc |
|  | Bbr_1381-rev | aagctgaggaccagtccgacg |
|  | Bbr_1828-fw | cgcttatcccgctcggcaaac |
|  | Bbr_1828-rev | gggctggattacgcgcgac |
|  | Bbr_0133-fw | gcgtgccctattgccgatgc |
|  | Bbr_0133-rev | gctgcaacgatgccacctcg |
|  | Bbr_0221-fw | cgattggcttggcttctacc |
|  | Bbr_0221-rev | gaatccgactacgaacagcg |
|  | Bbr_0222-fw | ctccatggctgtttcgatgg |
|  | Bbr_0222-rev | ctgaatcgtccttcttggcg |
|  | Bbr_0223-fw | tgcttgaacaattcgtggca |
|  | Bbr_0223-rev | tagttgacgaatgtgcgctg |
|  | Bbr_0224-fw | agaacctgaagctctccacc |
|  | Bbr_0224-rev | ctcgtcaccggaatcgtaga |
|  | Bbr_0225-fw | cagtcgtcctcaagccaaac |
|  | Bbr_0225-rev | gatgatcgaatcctcgctgc |
|  | Bbr_0226-fw | gctgtggaaaacgtgatggt |
|  | Bbr_0226-rev | cagaccaactttctccagcg |
| qRT-PCR control primer pairs | atpD-fw | cgtatgccttccgccgtgggttac |
|  | atpD-rev | acgtagatggcttgcagcgaggtg |
|  | rpoB-fw | cacgatggtgctgcgaccttccc |
|  | rpoB-rev | gacctgacggatacgacggttgcc |
|  | ldh-FW | gtgatgggcgagcatggcgactc |
|  | ldh-rev | ggaggcgaagcggtcttggttggtc |
|  | pdxS-fw | gatcaagggcattcaggaag |
|  | pdxS-rev | cgaactggttcttgtcgatc |
|  | gluC-fw | cttcgccacgaacttcttct |
|  | gluC-rev | gttcttgagcagtgcgatca |

^a^ Restriction sites incorporated into oligonucleotide primer sequences are indicated in bold.
